# NEONATAL SEPSIS: MORTALITY IN A MUNICIPALITY IN SOUTHERN BRAZIL, 2000 TO 2013

**DOI:** 10.1590/1984-0462/;2018;36;2;00001

**Published:** 2018-01-08

**Authors:** Jakeline Barbara Alves, Flávia Lopes Gabani, Rosângela Aparecida Pimenta Ferrari, Mauren Teresa Grubisich Mendes Tacla, Arnildo Linck

**Affiliations:** a Universidade Estadual de Londrina, Londrina, PR, Brasil.

**Keywords:** Sepsis, Neonatal mortality, Risk factors, Newborn Infant, Sepse, Mortalidade neonatal, Fatores de risco, Recém-nascido

## Abstract

**Objective:**

To describe the neonatal mortality coefficient attributed to sepsis and other causes, and to report the maternal, neonatal and death characteristics of newborn infants that died in the city of Londrina, Paraná, in Southern Brazil.

**Methods:**

This is a cross-sectional study with a time series analysis. Neonatal deaths that contained neonatal sepsis records in any field of the death certificate between the years 2000 and 2013 were studied. The years were grouped into biennia, and cause specific neonatal mortality coefficient was calculated, according to the International Classification of Diseases, 10th revision. Results are expressed as prevalence ratio and 95% confidence interval (95CI%). For bivariate analysis, p<0.05 was considered significant.

**Results:**

Among the 745 deaths, 229 (30.7%) had sepsis, with a neonatal mortality coefficient of 7.5 per one thousand livebirths. Sepsis was involved in 2.3 deaths per 1,000 live births. The main underlying causes were conditions originated in the perinatal period and congenital malformations. Sepsis was associated with pre-eclampsia, urinary tract infection, Apgar in the 1st and 5th minutes, and occurrence of late death. In the descriptive trend analysis, there was an increased proportion of mothers aged 35 years or older and with eight or more schooling years. Prenatal coverage was high, but a little more than half of the mothers attended seven or more medical appointments.

**Conclusions:**

In the 14 years analyzed, the prenatal care was identified as a preventive measure against maternal and fetal disorders and the advanced maternal age was associated with neonatal mortality.

## RESUMO

**Objetivo:**

Descrever o coeficiente de mortalidade neonatal por sepse e outras causas, além das características maternas, gestacionais, do parto, do recém-nascido e do óbito em Londrina, Paraná.

**Métodos:**

Estudo transversal e de séries temporais. Foram estudados óbitos neonatais que continham, em qualquer campo da declaração de óbito, registro de sepse neonatal, entre 2000 e 2013. Os anos foram agrupados em biênios e realizou-se cálculo do coeficiente de mortalidade neonatal e por causas específicas, segundo 10ª revisão da Classificação Internacional de Doenças (CID-10). Para a análise bivariada, considerou-se p<0,05, com cálculo da razão de prevalência e intervalo de confiança de 95% (IC95%).

**Resultados:**

Dos 745 óbitos, em 229 (30,7%) registrou-se sepse, com coeficiente de mortalidade neonatal de 7,5 óbitos por mil nascidos vivos (NVs), estando a sepse envolvida em 2,3 óbitos por mil NVs. As causas básicas da mortalidade neonatal foram afecções originadas no período perinatal e malformações congênitas. A sepse associou-se a pré-eclâmpsia, infecção do trato urinário, Apgar no 1º e 5º minutos e ocorrência de óbito tardio. Na análise descritiva de tendência, destacou-se o aumento na proporção de mães com 35 anos ou mais e com oito ou mais anos de estudo. A cobertura de pré-natal foi elevada, porém pouco mais da metade das mães realizou sete ou mais consultas.

**Conclusões:**

Nos 14 anos estudados, destacam-se o papel do pré-natal como ação preventiva dos agravos maternos e fetais e o aumento da idade e da escolaridade materna associados com a mortalidade neonatal.

## INTRODUCTION

The infant mortality coeficiente (death of infants aged less than 1 year per one thousand live births - LBs) is a sensitive indicator of social and economic development, and especially of health care in a specific geographic space and time. Even though the global mortality rate in childhood (children aged less than five years) has decreased 49% between 1990 and 2013 - from 90 to 46 deaths per one thousand LBs -, 74% of these deaths corresponded to children aged less than 1 year, and 44% occurred in the neonatal period (zero to 27 days of life).^1^

In the same period, the global neonatal mortality rate had a 40% reduction.^1^ In Brazil, the reduction in this coefficient is also present, decreasing from 16.7 to 10.6 deaths per one thousand LBs between 2000 and 2011. The South Region presented with rates lower than the national ones, showing 7.8 neonatal deaths per one thousand LBs in 2011.^2^ Even if the Brazilian rates are better, it is estimated that 60% of the deaths of children aged less than one year are neonatal.^3^

In 2013, the main causes of neonatal deaths in the world were complications resulting from premature labor (35%) and labor (24%), as well as those attributed to sepsis (15%), considered one of the main causes of death in this age group in Brazil.^3^ ^,^ ^4^ Sepsis is an organic dysfunction caused by a systemic inflammatory response resulting from one or more infection focuses in the body; there is a tissue cell lesion caused by the infection agent, and, depending on the genetic and physiological characteristics of the infant and the capacity of invasion, injury and pathogenicity of the agent, the infection can be generalized, with risk of shock and death.^5^ ^,^ ^6^

In a hospital in Santa Catarina, early sepsis contributed with the neonatal mortality rate: 50.3 cases per one thousand LBs.^7^ In 2007, in a maternity ward in the Amazon, the chance of death in infants increased 90 times among those with early sepsis.^8^ Freitas *et al.* identified that 68.2% of the premature infants who died in a hospital of Minas Gerais had late sepsis.^9^ According to data from the 15^th^ Health Regional of Paraná, the conditions in the perinatal period represented 54.8% of the neonatal deaths. Death by sepsis was considered reducible in 9.2% of the cases with early diagnosis and treatment, and education in health.^10^

Therefore, neonatal sepsis predisposes damage to the physical health of the newborn, with higher risk of death, besides being expensive for the health system, due to the demand for broad-spectrum antibiotic therapy, prolonged time of hospitalization and the need for invasive and highly-complex procedures. In this context, this study aimed at identifying the general neonatal mortality coefficient (NMC) and NMC with sepsis involved, besides revealing the causes and maternal, pregnancy, labor, newborn and death characteristics with the involvement of sepsis in a city in Southern Brazil. The objective is to clarify its descriptive profile between 2000 and 2013.

## METHOD

This is a cross-sectional, time series study, based on a larger project called “Determinants of infant mortality in the city of Londrina, Paraná”, whose data collection took place from 2000 to 2013. The city of Londrina is located in the North of the State of Paraná, with population estimated in 515,707 inhabitants, in 2012, of which 6,935 (1.3%) are children aged less than one year, and 25.774 (5.0%) are aged between one and four years.

The choice was to study newborns (zero to 27 days of life) who presented with medical diagnosis of neonatal sepsis in any blank of the death declaration (DD), when it is a basic, intermediate or immediate cause of death, and the File of Infant Death Investigation from the Municipal Committee of Prevention of Maternal and Infant Mortality (documents found in the files of the Center of Information and Mortality, from the Municipal Secretariat of Health in Londrina). The data were collected by Nursing students, previously trained for this purpose, attending Universidade Estadual de Londrina (UEL).

The variables were classified and studied according to the characteristics:

1. Maternal variables: maternal age (<35 years and ≥35 years); number of pregnancies (primigravid and multigesta); smoking (yes and no); alcoholism (yes and no); marital status (with and without a partner) and schooling (<8 years and ≥8 years);
2. Pregnancy and labor variables: conduction of the prenatal test (yes and no); number of prenatal appointments (<7 and ≥7); type of pregnancy (single and multiple); type of labor (C-section and vaginal); and maternal conditions (yes and no) - pre-eclampsia, gestational diabetes, maternal urinary tract infection; premature rupture of membranes and premature labor;
3. Neonatal and death variables: neonatal asphyxia (yes and no); gestational age (IG - <37 and ≥37 weeks); weight at birth (<2500 and ≥2500 g); Apgar score in the 1st and 5th minutes (<7 and ≥7); need for hospitalization after birth (yes and no); need for hospitalization in neonatal intensive care unit (ICU) (yes and no); type of neonatal death (early until six days of life, or late, from seven to 27 days of life).

Neonatal deaths were distributed in biennia aiming at the stabilization of rates, and the basic cause of death found in the DD was organized according to a chapter in the 10^th^ revision of the International Classification of Diseases (ICD-10). For the calculation of NMC, the number of deaths of children aged from zero to 27 days (numerator) was divided by the total number of LBs in the same city and year (denominator), times one thousand.

For the calculation of NMC for specific causes, the numerator was replaced with newborns who presented, in the DD, conditions originated in the perinatal period, congenital malformations, external causes of morbidity and mortality, and with sepsis involved. The term “sepsis involved”, in this study, means that, at some point, the newborn presented with a medical diagnosis of sepsis in the process of sickness and death, and this morbidity is present in any line of the DD, which indicates its involvement. Its analysis was made separately to demonstrate the behavior of the deaths with and without the involvement of sepsis, and to establish the main causes of death throughout the years.

NMC for a specific cause = deaths of children aged from 0 to 27 days for a specific cause x 1,000 LBs in the same city and year.

A bivariate analysis of the independent variables with the dependent variable was conducted (being involved or not of sepsis in the proves of sickness and death), using the chi-square test with Yeates’s correction, considering p<0.05 as significant, calculating the prevalence ratio (PR) and the 95% confidence interval (95%CI).

The data were analyzed using the IBM Statistical Package for the Social Sciences (SPSS)^®^, version 19,0 (US). The primary research was submitted to the appreciation of the Health Care Board of the Municipal Autarchy of Health in Londrina, and approved by the Research Ethics Committee of the Nursing School at Universidade de São Paulo (CEP-EEUSP).

## RESULTS

From 2000 to 2013, there were 745 neonatal deaths, of which 229 (30.7%) had a medical diagnosis of sepsis registered in any field of the DD. The NMC was of 7.5 deaths per one Thousand LBs; the neonatal mortality with involvement of sepsis was of 2.3 deaths per one thousand LBs.

The most prevalent causes of death, their representation by NMC and the biennia with higher coeficiente in 14 years were, respectively: conditions originated in the perinatal period, 7.4 deaths per one thousand LBs in the biennia 2006-2007; congenital malformations, 1.7 deaths per one thousand LBs in the biennia 2008-2009; and external causes of morbidity and mortality, 0.2 deaths per one thousand LBs in the biennia 2006-2007. The highest NMC with involvement of sepsis was 2.97 deaths per one thousand LBs in 2006-2007.

Higher frequencies of neonatal sepsis in deaths stood out according to the ICD-10 in the conditions originated in the perinatal period (197; 86.0%) and congenital malformations (29; 12.7%). Other causes, also classified according to the ICD-10, included infectious and parasitic diseases, neoplasms and conditions of the respiratory system (1; 0.4% each chapter). The immediate causes of neonatal deaths present in the DD were: events related directly or indirectly with sepsis (170; 74.2%), neonatal infection (18; 7.9%), hemorrhagic conditions of the neonatal period (18; 7.9%), metabolic and hydroelectrolytic diseases (6; 2.6%), conditions of the central nervous system (5; 2.2%), respiratory diseases (5; 2.2%), and others (7; 3.0%).

In the association of the independent variables with the outcome (Table 1), there was statistical significance in the association of deaths with sepsis and pre-eclampsia (PR 1.47; 95%CI 1.15-1.87), urinary tract infection during pregnancy (PR 1.42; 95%CI 1.13-1.79), Apgar at 1 minute <7 (PR 0.56; 95%CI 0.45-0.69), Apgar at 5 minutes <7 (PR 0.41; 95%CI 0.31-0.53) and occurrence of late death (PR 3.42; 95%CI 2.78-4.20).

**Table 1:** Analytical distribution of neonatal deaths with medical diagnosis of sepsis, 2000-2013. Londrina (PR).

Variable*	n known	n unknown	%	p-value	PR and 95%CI
Maternal age ≥35 years	211	18	31.5	0.85	1.03 (0.79-1.39)
Primipregnancy	208	21	33.3	0.10	1.20 (0.96-1.49)
Maternal smoking	203	26	31.8	0.69	1.05 (0.80-1.40)
Maternal alcoholism	203	26	40.4	0.13	1.35 (0.94-1.95)
Mother with partner	211	18	314	0.34	0.85 (0.62-1.18)
Maternal schooling >8 years	211	18	30.1	0.54	1.07 (0.85-1.36)
Prenatal appointment	210	19	31.4	0.10	0.66 (0.38-1.13)
N. of prenatal appointments <7	200	29	28.7	0.19	0.86 (0.68-1.07)
Single pregnancy	200	29	31.8	0.08	0.74 (0.52-1.05)
C-section	211	18	33.7	0.07	1.22 (0.98-1.51)
Pre-eclampsia	182	47	42.3	<0.01	1.47 (1.15-1.87)
Gestational diabetes	179	50	43.5	0.22	1.39 (0.86-2.24)
Urinary tract infection	182	47	27.0	<0.01	1.42 (1.13-1.79)
Premature rupture of membranes	178	51	27.7	0.29	0.85 (0.63-1.15)
Premature labor	181	48	29.5	0.08	0.79 (0.62-1.02)
Neonatal asphyxia	162	67	25.3	0.31	0.83 (0.57-1.20)
Gestational age <37 weeks	211	18	31.4	0.47	1.10 (0.83-1.45)
Weight at birth <2500 g	211	18	31.1	0.71	1.05 (0.80-1.37)
Apgar at 1 minute<7	208	21	26.0	<0.001	0.56 (0.45-0.69)
Apgar at 5 minutes <7	208	21	17.6	<0.001	0.41 (0.31-0.53)
Hospitalization after birth	194	35	31.2	0.70	0.93 (0.66-1.31)
Need for neonatal ICU	155	74	31.6	0.74	0.90 (0.51-1.61)
Late death (7 to 27 days of life)	211	18	63.2	<0.001	3.42 (2.78-4.20)

*Excluding records with ignored information; ICU: intensive care unit.

Table 2 shows the distribution of neonatal deaths in which there was involvement of sepsis, acccording to the maternal characteristics. The frequency of death was higher among mothers aged less than 35 years, ranging from 76.2 to 93.9%, however, higher proportions between children of mothers aged more than 35 years occurred in the biennia 2006-2007 (20.0%) and 2012-2013 (23.8%). The newborns of primigravida mothers presented higher percentage of death, especially in the biennia 2000-2001 (68.8%), and 2002-2003 (68.2%). In 2006 and 2007, neonatal death prevailed among multiple pregnancies (62.5%).

**Table 2:** Distribution of neonatal deaths with sepsis, according to maternal characteristics, in biennia 2000-2013. Londrina (PR).

	Biennia of death*														Total	
	2000 2001		2002 2003		2004 2005		2006 2007		2008 2009		2010 2011		2012 2013			
	n	%	n	%	n	%	n	%	n	%	n	%	n	%	n	%
Maternal age (years)																
<35	28	87.5	19	86.4	22	84.6	32	80.0	30	88.2	31	93.9	32	76.2	194	84.7
≥35	4	12.5	3	13.6	4	15.4	8	20.0	4	11.8	2	6.1	10	23.8	35	15.3
Number of pregnancies																
Primigravid	22	68.8	15	68.2	14	53.8	15	37.5	19	55.9	20	60.6	23	59.0	128	56.6
Multigesta	10	31.3	7	31.8	12	46.2	25	62.5	15	44.1	13	39.4	16	41.0	98	43.4
Smoking																
Yes	8	25.0	4	18.2	6	23.1	7	17.5	6	17.6	4	13.8	6	15.8	41	18.6
No	24	75.0	18	81.8	20	76.9	33	82.5	28	82.4	25	86.2	32	84.2	180	81.4
Alcoholism																
Yes	3	9.4	-	-	1	3.8	4	10.0	5	14.7	2	6.9	4	10.5	19	8.6
No	29	90.6	22	100	25	96.2	36	90.0	29	85.3	27	93.1	34	89.5	202	91.4
Marital status																
With a partner	24	75.0	18	81.8	23	88.5	34	85.0	29	85.3	30	90.9	40	95.2	198	86.5
Without a partner	8	25.0	4	18.2	3	11.5	6	15.0	5	14.7	3	9.1	2	4.8	31	13.5
Schooling (years)																
Up to 7	14	43.8	5	22.7	7	26.9	15	37.5	6	17.6	9	27.3	12	28.6	68	29.7
≥8	18	56.3	17	77.3	19	73.1	25	62.5	28	82.4	24	72.7	30	71.4	161	70.3

*Excluding records with ignored information.

As to maternal smoking and alcoholism in deaths associated with sepsis, smoking presented higher proportion in biennia 2000-2001 (25.0%), and 2004-2005 (23.1%), with progressive reduction of about 10.0% after this last biennia; alcoholism remained irregular during the analyzed years. The frequency of neonatal death with involvement of sepsis remained high among mothers with a partner, and showed progressive increase throughout the years, ranging from 75.0 to 95.2%. As to schooling, the highest percentages of death related with sepsis were concentrated among mothers who had eight or more schooling years, and stood out in the biennia 2008-2009, with 82.4% of the cases.

The gestational and labor characteristics of the children who presented sepsis involved in death can be observed in Table 3. The frequency of prenatal was high in all biennia (90.6 to 100.0%), but only in 2012 and 2013 there was a higher frequency of pregnant women with seven or more prenatal appointments (52.8%). Single pregnancies were also prevalent in all years (80.8 to 97.0%), but the highest frequency of multiple pregnancies occurred in 2004 and 2005 (19.2%). Vaginal labor was prevalent in relation to the C-section only in biennia 2000-2001 (56.3%) and 2006-2007 (52.5%). Regarding the intercurrences in pregnancy, pre-eclampsia (6.5 to 43.2%), urinary tract infection during pregnancy (29.0 to 62.2%) and premature labor (35.7 to 88.5%) stand out.

**Table 3:** Distribution of neonatal deaths with sepsis, according to gestational and labor characteristics, in biennia 2000-2013. Londrina (PR).

	Biennia of death*														Total	
	2000 2001		2002 2003		2004 2005		2006 2007		2008 2009		2010 2011		2012 2013			
	n	%	n	%	n	%	n	%	n	%	n	%	n	%	n	%
Prenatal care																
Yes	29	90.6	22	100	26	100	37	92.5	32	94.1	32	97.0	39	95.1	217	95.2
No	3	9.4	-	-	-	-	3	7.5	2	5.9	1	3.0	2	4.9	11	4.8
N. of prenatal appointments																
<7	24	75.0	12	54.5	14	53.8	31	77.5	17	50.0	16	57.1	17	47.2	131	60.1
≥7	8	25.0	10	45.5	12	46.2	9	22.5	17	50.0	12	42.9	19	52.8	87	39.0
Type of pregnancy																
Single	29	90.6	20	90.9	21	80.8	33	82.5	30	88.2	32	97.0	27	87.1	192	88.1
Multiple	3	9.4	2	9.1	5	19.2	7	17.5	4	11.8	1	3.0	4	12.9	26	11.9
Type of labor																
C-section	14	43.8	14	63.6	19	73.1	19	47.5	18	52.9	19	57.6	28	66.7	131	57.2
Vaginal	18	56.3	8	36.4	7	26.9	21	52.5	16	47.1	14	42.4	14	33.3	98	42.8
Pre-eclampsia																
Yes	2	6.5	7	33.3	9	34.6	16	43.2	5	15.6	8	34.8	11	36.7	58	29.0
No	29	93.5	14	66.7	17	65.4	21	56.8	27	84.4	15	65.2	19	63.3	142	71.0
Gestational diabetes																
Yes	-	-	1	4.8	3	11.5	2	5.4	1	3.1	1	4.5	2	7.1	10	5.1
No	31	100	20	95.2	23	88.5	35	94.6	31	96.9	21	95.5	26	92.9	187	94.9
Urinary tract infection during pregnancy																
Yes	9	29.0	9	42.9	13	50.0	23	62.2	13	40.6	13	54.2	13	44.8	93	46.5
No	22	71.0	12	57.1	13	50.0	14	37.8	19	59.4	11	45.8	16	55.2	107	53.5
Premature rupture of membranes																
Yes	5	16.1	9	42.9	4	15.4	9	24.3	6	18.8	2	9.1	4	14.8	39	19.9
No	26	83.9	12	57.1	22	84.6	28	75.7	26	81.3	20	90.9	23	85.2	157	80.1
Premature labor																
Yes	24	77.4	16	76.2	23	88.5	32	86.5	25	78.1	10	41.7	10	35.7	140	70.4
No	7	22.6	5	23.8	3	11.5	5	13.5	7	21.9	14	58.3	18	64.3	59	29.6

*Excluding records with ignored information.

The specific characteristics of the newborn and of the death of newborns who presented with involvement of sepsis are in Table 4. Neonatal asphyxia remained high in deaths with sepsis, presenting higher proportion in the biennia 2002-2003 (88.9%). Both the low weight at birth and prematurity presented higher percentages of death with sepsis in the period analyzed, especially in 2004 and 2005, with identical frequency of 88.5% for both variables. Low Apgar at 1 minute score (<7) was frequent in all biennials, except in 2004 and 2005 (50.0%). Most newborns analyzed required hospitalization after birth (77.8 to 95.7%), especially in a neonatal ICU environment (85.2 to 97.0%). Regarding the period of death occurrence, deaths were prevalent after the sixth day of life, characterizing late neonatal death, except for the biennia 2008-2009, when there was no difference in percentage (50.0%).

**Table 4:** Distribution of neonatal deaths with sepsis, according to characteristics of the newborn and death, in biennia 2000-2013. Londrina (PR).

	Biennia of death*														Total	
	2000 2001		2002 2003		2004 2005		2006 2007		2008 2009		2010 2011		2012 2013			
	n	%	n	%	n	%	n	%	n	%	n	%	n	%	n	%
Neonatal asphyxia																
Yes	17	68.0	16	88.9	12	57.1	18	54.5	20	71.4	17	63.0	19	67.9	119	66.1
No	8	32.0	2	11.1	9	42.9	15	45.5	8	28.6	10	37.0	9	32.1	61	33.9
Gestational age (weeks)																
<37	27	84.4	18	81.8	23	88.5	31	77.5	25	73.5	26	78.8	35	83.3	185	80.8
≥37	5	15.6	4	18.2	3	11.5	9	22.5	9	26.5	7	21.2	7	16.7	44	19.2
Weight at birth (g)																
<2500	23	71.9	19	86.4	23	88.5	33	82.5	24	70.6	28	84.8	32	76.2	182	79.5
≥2500	9	28.1	3	13.6	3	11.5	7	17.5	10	29.4	5	15.2	10	23.8	47	20.5
Apgar at 1 minute																
<7	21	65.6	12	54.5	13	50.0	24	64.9	24	70.6	22	66.7	28	66.7	144	63.7
≥7	11	34.4	10	45.5	13	50.0	13	35.1	10	29.4	11	33.3	14	33.3	82	36.3
Apgar at 5 minutes																
<7	9	28.1	3	13.6	4	15.4	10	27.0	9	26.5	12	36.4	14	33.3	61	27.0
≥7	23	71.9	19	86.4	22	84.6	27	73.0	25	73.5	21	63.6	28	66.7	165	73
Hospitalization after birth																
Yes	25	89.3	19	86.4	22	95.7	34	89.5	30	93.8	28	84.8	28	77.8	186	87.7
No	3	10.7	3	13.6	1	4.3	4	10.5	2	6.3	5	15.2	8	22.2	26	12.3
Hospitalization in neonatal ICU																
Yes	22	95.7	14	93.3	21	100	32	97.0	26	100	27	96.4	23	85.2	165	95.4
No	1	4.3	1	6.7	-	-	1	3.0	-	-	1	3.6	4	14.8	8	4.6
Type of death																
Early	10	31.3	9	40.9	12	46.2	18	45.0	17	50.0	14	42.4	20	47.6	100	43.7
Late	22	68.8	13	59.1	14	53.8	22	55.0	17	50.0	19	57.6	22	52.4	129	56.3
Basic cause																
Perinatal conditions	28	87.5	18	81.8	23	88.5	36	90.0	29	85.3	27	81.8	36	85.7	197	86.0
Malformations	4	12.5	4	18.2	3	11.5	4	10.0	4	11.8	5	15.2	5	11.9	29	12.7

*Excluding records with ignored information; ICU: Intensive care unit.

## DISCUSSION

This study aimed at identifying the NMC, its causes and the maternal, gestational, labor, newborn and death aspects of neonatal deaths with involvement of sepsis in Londrina (PR), between 2000 and 2013. 

The basic causes of death that were most identified were conditions originated in the perinatal period, resulting from congenital malformations, similarly to the findings of a study conducted in the 15^th^ Health Regional of Parana during a six-year period.^10^ The CMN in that study was lower (7.5/1,000 LBs) than that found in a hospital in Venezuela (16.11/1,000 LBs), and close to that identified in the region of São Paulo, with minimum coeficiente of 6.33 deaths per 1,000 LBs.^11^ ^,^ ^12^ The rate in the city of Londrina is in accordance with the South Region of the country (7.8/1,000 LBs), but it was lower in comparison to the State of Paraná (8.3/1,000 LBs) and Brazil (10.6/1,000 LBs) in 2011.^2^ On the other hand, the rate found in the State of Mexico was even lower, with 4.2 deaths per 1,000 LBs.^13^

Considering the maternal aspects, the trend in the number of neonatal deaths with sepsis among mothers aged more than 35 years corroborates a study in which the advanced maternal age had an influence on the development of early neonatal sepsis.^14^ Advanced maternal age predisposes the woman to higher incidence of problems, with consequent neonatal repercussion.

Maternal smoking, considered as a risk factor for prematurity, also increases the probability of newborns acquiring infections, being born with low weight and low Apgar score.^15^ ^,^ ^16^ In this study, the higher frequencies of premature deaths with involvement of sepsis among smoking mothers occurred in the biennia 2000-2001 and 2004-2005. Maternal alcoholism, on the other hand, favors the occurrence of conditions such as restriction of intrauterine growth and low weight at birth, which are characteristics observed in deaths by sepsis^17^ ^,^ ^18^; in this study, the highest percentage of alcoholism occurred in the biennia 2008-2009. Regarding marital status, a cohort of the Brazilian macro-regions performed in 2014 identified that being pregnant without a partner is a risk factor for neonatal mortality.^19^ In this study, there was no significant difference between deaths with or without sepsis, according to marital status. About maternal schooling, Benincá *et al.* pointed out that neonatal deaths by sepsis were more present in newborns of mothers with low schooling; however, as in this study, there is an inversion in this finding due to the higher frequency of neonatal deaths with sepsis among mothers with more schooling years. Maternal schooling was not associated with the development of sepsis in this study; however, the increasing frequency in the past years may be attributed to the higher frequency of women in the work market, followed by the intensive need for professional qualification for this purpose.^20^ In Ethiopia, a study identified 56% fewer chances of neonatal death with higher maternal schooling.^21^

The coverage of prenatal appointments was high in this study, above 90% in all biennials, reaching 100% between 2002 and 2005. However, the frequency of appointments became the focus of attention, reaching a little more than 50%, when considering the minimum of seven. The lowest concentration of prenatal appointments may have resulted in the highest incidence of premature labors throughout the years. When the prenatal follow-up is not satisfactory, the chances of sepsis may increase from two^22^ to tem times.^8^

Unlike this paper, a study shows higher frequency of neonatal deaths by sepsis in multiple pregnancies, when compared to single pregnancies.^20^ As to type of labor, the highest prevalence of C-sections was observed in China, and, in this study, it was a statistically significant variable for deaths of newborns with sepsis.^14^ In Santa Catarina, the mortality coefficient by sepsis was higher in C-sections than in vaginal labor, which leads to the consideration that cases of neonatal sepsis may be influenced by the high rates of C-sections in Brazil.^20^

About maternal problems, pre-eclampsia contributed with neonatal mortality; gestational diabetes is related to the higher incidence of prematurity.^23^ ^,^ ^24^ The maternal urinary tract infection was also present in a study conducted in Santa Catarina, in which 37% of the newborns with sepsis were children of pregnant women with this intercurrence in the cycle.^7^ Both pre-eclampsia and the urinary tract infection were significantly associated with deaths by sepsis in this study. In Acre, a study conducted about pregnancies with premature rupture of the membranes showed that 18.6% of the newborns presented with sepsis, and similar data were found in other analyses.^14^ ^,^ ^25^ ^,^ ^26^ ^,^ ^27^

In this study, regarding the newborn, neonatal asphyxia presented high frequency of occurrence in deaths with sepsis, however, without statistical relevance. According to Pinheiro *et al.*, this condition may increase in about 20 times the chances of developing neonatal sepsis.^8^ Regarding gestational age and weight at birth, in an ICU of Minas Gerais, 68.2% of the newborns who were diagnosed with sepsis had death as an outcome, with increasing prevalence in premature infants and newborns underweight.^9^ Pinheiro *et al.* showed that the chances of a newborn developing neonatal sepsis increases 21 times in cases of low weight at birth. Rugolo *et al.* showed, in a study, that 23.7% of the premature infants with very low weight in Brazilian university hospitals developed late-onset sepsis, whereas 22.9% presented clinical signs of this condition.^8^ ^,^ ^28^

Regarding the Apgar score, there was lower prevalence of sepsis in newborns whose score was lower than seven, both in the 1^st^ and in the 5^th^ minutes. However, in the calculation of PR, the numbers found were lower to one. Therefore, it is possible to question the low Apgar score as something positive for the newborn regarding death with sepsis involved. This observation is associated with the fact that these newborns receive more care in the perinatal period, in the labor room, with a strict follow-up of birth conditions; and then, in the hospitalization units, where they are monitored for several parameters. According to Mohaddesi *et al.*, 51.6% of the newborns who died between 2007 and 2009 had Apgar score lower than six in the 1st minute, with chances of death in this age group being six times higher.^29^

This study showed higher frequency of hospitalization, including the need for neonatal ICU care, of newborns diagnosed with sepsis. Zamudio *et al.* verified that sepsis is the main cause of death in premature infants, besides being the second cause of hospitalization in a hospital in Mexico.^13^ Olusanya pointed out neonatal sepsis as a factor for hospitalization in 2013, in Nigeria.^30^ In a tertiary hospital in Paraná, 49.1% of the cases of sepsis occurred secondarily to hospital infection, and hospitalization was an aggravating factor, as well as the need for invasive procedures, especially orotracheal intubation.^31^

Late neonatal death was the most prevalent in this study, with statistical significance. Sepsis increases the risk of death in six times, besides increasing the permanence in a neonatal ICU, prolonging the survival and favoring deaths after the first week of life.^30^

## CONCLUSIONS

Even though this is a cross-sectional study, in which it is not possible to establish a causal relationship between the factor and the outcome, this study aimed at filling out the gap in the state of the art about the proposed subject, aiming at showing the descriptive behavior of some independent variables throughout the years. Besides, the idea is to detail the specific mortality coefficient by cause, allowing to compare with other national and international rates using the data base referring to 14 years of study.

Although this is a descriptive study referring to a specific city, with data susceptible to human error, the large sample and the long period analyzed stand out, allowing a deep analysis of the theme. It was also possible to contribute with the strengthening of data previously published about the factors involved with sepsis in the studied age group, and the study adds new perspectives as to maternal age and schooling, as well as the development of problems in the neonatal period.

In the bivariate analysis, it was possible to mention variables that require more theoretical and analytical observations, associated with neonatal sepsis in further studies, especially of longitudinal design.
